# Comparative Metagenomics Reveal Phylum Level Temporal and Spatial Changes in Mycobiome of Belowground Parts of *Crocus sativus*

**DOI:** 10.1371/journal.pone.0163300

**Published:** 2016-09-29

**Authors:** Sheetal Ambardar, Heikham Russiachand Singh, Malali Gowda, Jyoti Vakhlu

**Affiliations:** 1 School of Biotechnology, University of Jammu, Jammu, India; 2 Next Generation Genomics Facility, C-CAMP, NCBS, Bangalore, India; University of Wisconsin Madison, UNITED STATES

## Abstract

Plant-fungal associations have been explored by routine cultivation based approaches and cultivation based approaches cannot catalogue more than 5% of fungal diversity associated with any niche. In the present study, an attempt has been made to catalogue fungal diversity associated with belowground parts i.e. rhizosphere and cormosphere, of *Crocus sativus* (an economically important herb) during two growth stages, using cultivation independent ITS gene targeted approach, taking bulk soil as reference. The 454 pyrosequencing sequence data analysis suggests that the fungal diversity was niche and growth stage specific. Fungi diversity, in the present case, was not only different between the two organs (roots and corm) but the dominance pattern varies between the cormosphere during two growth stages. *Zygomycota* was dominant fungal phylum in the rhizosphere whereas *Basidiomycota* was dominant in cormosphere during flowering stage. However in cormosphere though *Basidiomycota* was dominant phylum during flowering stage but *Zygomycota* was dominant during dormant stage. Interestingly, in cormosphere, the phyla which was dominant at dormant stage was rare at flowering stage and vice-versa (*Basidiomycota*: Flowering *=* 93.2% Dormant = 0.05% and *Zygomycota*: Flowering = 0.8% Dormant = 99.7%). At genus level, *Rhizopus* was dominant in dormant stage but was rare in flowering stage (*Rhizopus*: Dormant = 99.7% Flowering = 0.55%). This dynamics is not followed by the bulk soil fungi which was dominated by *Ascomycota* during both stages under study. The genus *Fusarium*, whose species *F*. *oxysporum* causes corm rot in *C*. *sativus*, was present during both stages with slightly higher abundance in roots. Interestingly, the abundance of *Rhizopus* varied a great deal in two stages in cormosphere but the abundance of *Fusarium* was comparable in two growth stages (Bulk soil Flowering = 0.05%, Rhizosphere Flowering = 1.4%, Cormosphere Flowering = 0.06%, Bulk soil Dormant = 2.47% and cormosphere dormant = 0.05%). This is the first report on the fungal diversity associated with the root of *Crocus sativus* and first report on the fungi associated with corm of any plant with the temporal and spatial variation in the fungal community structure.

## Introduction

Plant associated microbial community influence plants, as it promotes their growth, increases stress tolerance and mediates local patterns of nutrient cycling [1, 2]. Plant- microbe interactions occur in rhizosphere [2, 3, 4], phyllosphere [5], endosphere [6], carposphere [7] and cormosphere [4, 8]. The microbes mostly studied in association with plants by cultivation-dependent as well as cultivation-independent approaches are rhizobacteria [1–4, 6, 9–11]. Plant associated fungal interactions based on cultivation independent metagenomic approach are under-represented in literature and the routine cultivation dependent approaches to study plant -fungal associations are dominated by endophytic and arbuscular mycorrhizal fungi [6, 12–19]. Previously studies on saffron–fungal associations have been done mostly on fungal endophytes and the bioactive compounds of these endophytes [15–17]. Since only <5% of the fungi can be retrieved by routine laboratory cultivation dependent techniques [20], the complete fungal diversity can only be assessed by the cultivation-independent metagenomic approaches [10, 21–29]. Cultivation-independent metagenomic methods based on cloning approaches (metagenomic library based), generally underestimate microbial diversity as it suffers from an inherent cloning bias. The representation of microbial diversity is further restricted by the total number of clones selected for sequencing [19, 30–32]. Results of diversity and community ecology studies, strongly depend on unbiased sampling depth which can be attained by high throughput next generation sequencing, a cloning-independent direct metagenomic sequencing approach. Direct sequencing eliminates bias resulting from cloning and enables extensive sequencing of microbial populations resulting in better representation of microbial diversity in various ecological niches [7, 21, 24, 26, 29, 33, 34].

*Crocus sativus* L. (Saffron) is economically important, as it is world’s highest priced (~4500 US$ per Kg) medicinal, aromatic plant and is referred as the ‘Golden Condiment’. It is an autumn blooming perennial herbaceous plant, whose activity slows down in spring in contrast to most flowering plants [35]. It is a sterile triploid (3n = 24) and propagates by underground vegetative organs known as corms. The herb has an interesting biannual life cycle that is characterized by three distinct stages dormant (July-Aug), flowering (Oct-Nov) and vegetative stage (Jan-May). Corms are sown in July-August and depending upon their weight, if more than 8g can flower in same year October-November or may go through the annual cycle to attain the minimum required weight to support flowering. Flowering stage is followed by emergence of grass like leaves in vegetative stage wherein daughter corms/cormlets are born and vegetative phase finally leads to the next dormant growth phase [35]. The study on the fungal diversity on the below ground parts of *C*. *sativus* has been initiated to test the hypothesis that fungal-root/corm associations are specific to particular niche and growth stages as reported for bacterial diversity associated with its below ground parts earlier [4]. Previously plant growth promoting rhizobacteria of *C*. *sativus* rhizosphere using cultivation-dependent approach and the bacterial diversity of rhizosphere, cormosphere and bulk soil using cultivation-independent 16S rRNA gene targeted metagenomics approach has been reported [4, 36]. In the present study, the dynamics of fungal diversity associated with the rhizosphere, cormosphere and the bulk soil have been unravelled during two growth stages by internal transcribed spacer (ITS) gene targeted metagenome sequencing approach. Microbial associations of roots of many plants have been investigated however the microbes associated with the belowground parts of plants such as corm in *Gladiolus*, tuber in sweet potato, bulbs in onion and garlic have not been studied till date. This is the first report on the fungi associated with corm (in addition to root) to understand the diversity and dynamics of fungi associated with belowground parts of the herb. The fungal diversity catalogued by metagenomic approach can be exploited for selective isolation of plant growth promoting fungi and their subsequent use for plant growth promotion in *C*. *sativus*.

## Materials and Methods

### Sample sites and collection method

Samples such as bulk soil, rhizosphere and cormosphere from the *C*. *sativus* were collected during the two growth stages [(dormant period-Aug 2010, flowering-Nov 2010) from Wuyan village (74°58′0″E, 34°1′30″N, 5173ft)] in Pulwama district of Kashmir, India. The soil sampling was done as per the protocol standardized by Luster and coworkers [37]. Composite rhizosphere and cormosphere samples were analyzed by collecting the samples from the four corners of three different fields and mixed together. The bulk soil was collected after vigorous shaking of the roots and the soil that remains adhered to the roots, was taken as rhizosphere soil. The corms sheath was taken to study corm-associated bacteria. The samples were collected in triplicates from the three corners of field and pooled together and were transported to the laboratory at 4°C (in ice) and stored at -20°C.

### PCR amplification of ITS gene

Metagenomic DNA was extracted from 1 g of bulk soil following the protocols given by Pang and co-workers [38].The same protocol was modified for extraction DNA from rhizosphere and cormosphere, wherein 1 g of roots and corms sheath was taken respectively for DNA extraction. The crude DNA extracted was further purified by gel elution kit (Macherey–Nagel, Nucleospin Extract II kit), analysed on 1% agarose gel and stored at -20°C.

Internal transcribed spacer region (ITS1-ITS4) was amplified using universal primer pair; ITS1F (5’-TCCGTAGGTGAACCTGCGG-3’) and ITS4R (5’-TCCTCCGCTTATTGATATGC-3’) [39] from metagenomic DNA samples. The PCR mixture contained 1–10 ng of DNA extracted from samples of *C*. *sativus*, 10 pM of universal primers, 1X PCR buffer (Fermentas), 2.5mM MgCl_2_, 2.5U of Taq DNA polymerase (Fermentas), 0.2mM each deoxynucleoside triphosphate (Fermentas) and sterile filtered MilliQ water to a final volume of 50 μl. Negative controls comprised of same assay without the template. PCR amplification was performed in a DNA thermocycler (Eppendorf, India) following the amplification program of initial denaturation at 94°C for 5 min, 30 cycles of 94°C for 30 sec, annealing at 55°C for 30 sec, and extension at 72°C for 1 min 30 sec, and a final extension of 10 min at 72°C. The amplicons of approximately 550 bp were analyzed by electrophoresis on 1% agarose gel and a 100 bp DNA ladder (Fermentas) was taken as the molecular size standard. The amplicon (~550 bp) was gel purified from all the five samples using a gel elution kit (Qiagen) and sent for pyrosequencing at Roche Diagnostics India Pvt. Ltd., New Delhi.

### Pyrosequencing of ITS amplicons

The pyrosequencing library was prepared by ligating ITS gene to unique Molecular Identifier Tags (MIDs). The amplified Internal Transcribed Spacer (ITS) region of five samples were attached to 5 unique MIDs that helped in sample identification when analyzed in parallel on a 454 picotiter plate and sequencing was performed with 454, GS Junior sequencer (454 Life Sciences, Brandford, USA).

### Bioinformatics analysis of sequences obtained

The 454 sequences obtained for fungal ITS gene were analysed using various bioinformatics softwares. The raw sequence data was demultiplexed based on sample-specific barcode tags. The primer and tag sequences were trimmed from sorted sequences. The sequences having quality score less than 20 and short sequences with a length less than 200 nucleotides were trimmed from the demultiplexed data. The resulting sequences were analysed by Mothur software [40]. Mothur is software package that can be used to trim, screen, and align sequences; calculate distances; assign sequences to operational taxonomic units (OTU); and describe α and β diversity, thus analyzing community sequence data characterized by pyrosequencing [40]. The ITS gene sequence (~550 bp) was analysed for their reference counterparts using Mothur pyrosequencing pipeline (software version 1.28.0) against the QIIME ITS fungal database using the Wang method of classification [40]. Rarefaction curves, venn diagram and heatmap were generated along with calculation of diversity indices, unifrac weighted significance, Libshuff and parsimony using pyrosequencing pipeline of Mothur. Rarefaction curves (97% identity) were plotted between the number of observed Operational Taxonomy Units (OTUs) (cluster count) and the number of reads of the samples. α diversity was determined by Shannon’s Diversity Index and Chao1 estimate. Venn diagram, Heatmap, weighted and unweighted UniFrac metrics were utilized to evaluate β diversity [41].

### GenBank Accession numbers

The sequences obtained in this study were submitted to nucleotide databases by NCBI-BankIt and available at the GenBank under accessions number SRR1535294.

## Results and Discussion

The dynamics of fungal diversity associated with the rhizosphere and cormosphere of *C*.*sativus* with the bulk soil as reference, during two growth stages has been studied using high throughput sequencing. The hypothesis put to test in this study is, as in bacterial diversity the fungal root/corm associations are niche specific with seasonal/temporal variations. The associated fungal diversity in the present study was assessed by gene targeted metagenomic approach, wherein the amplified ITS region from the total metagenomic DNA has been sequenced by 454 pyrosequencing technology (Roche Diagnostic, New Delhi). Sequencing of the amplicons yielded 89,527 reads (47,078,862 bp) with a median read length of 517bp. Following quality trimming (Q20), denoising and chimera removal, 22,358 ITS sequences were subjected to downstream analyses using pyrosequencing pipeline, Mothur [40]. The number of reads obtained per sample varied from 2068–8418 (Table 1). About 75% of the raw sequences were discarded in order to achieve good quality score i.e. Q20 to draw accurate conclusions. Lower Q score would have resulted in discard of less raw sequences but would have compromised on the quality of reads. Similar to present study, the variation in read numbers (970 to 12157) after the quality trimming (Q25) was observed in forest soil fungal diversity analysed using pyrosequencing by Shi and coworkers [42].

**Table 1:** Total number of fungal ITS sequences obtained per sample by pyrosequencing and its clustering at 97% similarity.

Sample	Raw reads	Quality Reads
BULK SOIL DORMANT	11,618	2068
BULK SOIL FLOWERING	5824	2,217
CORMOSPHERE DORMANT	20,884	4,179
CORMOSPHERE FLOWERING	18,986	8,418
RHIZOSPHERE FLOWERING	31,865	5,476
**Total**	**89,527**	**22,358**

The number of sequencing reads were maximum in rhizosphere and minimum in bulk soil during flowering stage.

**Fig 1:**
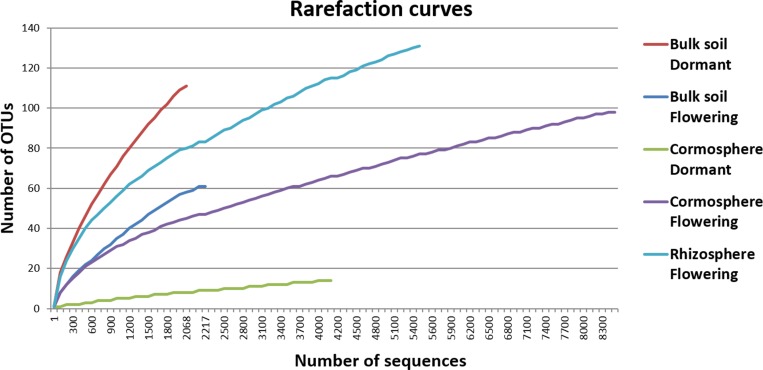
Rarefaction curves for fungal OTUs clustering at 97% rRNA sequence similarity from the five niches of *C*. *sativus*. Rarefaction curves represent more diversity in bulk soil during dormant stage as compared to rhizosphere and cormosphere. Curves represent sequences for bulk soil, cormosphere and rhizosphere during flowering and bulk and cormosphere during dormant stage of *C*. *sativus*.

**Fig 2:**
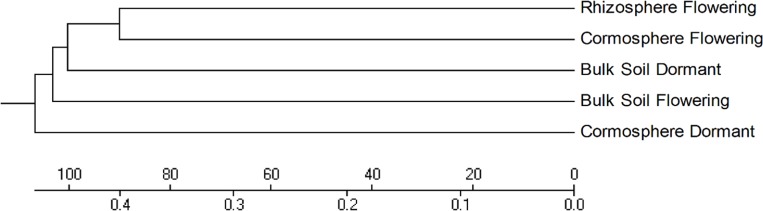
Jackknife dendrogram of fungal communities associated with bulk soil, cormosphere and rhizosphere during flowering stage and bulk soil and cormosphere during dormant stage of *C*. *sativus*. The Jackknife tree depicts that the rhizosphere is phylogenetically similar to cormosphere during flowering stage than other samples.

**Fig 3:**
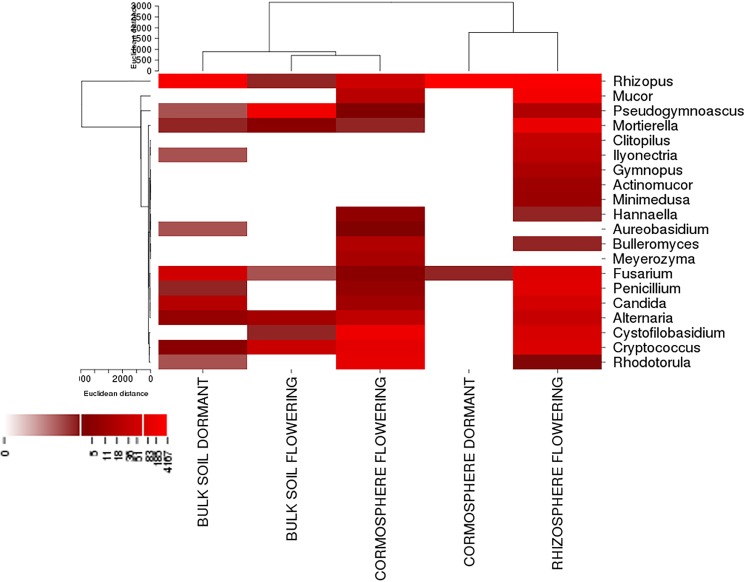
Heatmap of top 20 fungal genera of bulk soil, cormosphere and rhizosphere during flowering and bulk and cormosphere during dormant stage of *C*. *sativus*. The heatmap depicts the relative abundance of top 20 fungal genera (y axis) across the 5 samples analysed (x axis). The heatmap colors represent the relative abundance of fungal genera within each sample. Square colors shifted towards bright red indicate higher abundance. The relative abundance values of each genus for each sample are reported in S2 and S3 Tables. The heat map indicates dynamic shift in fungal diversity across belowground organs of plant and also at two different growth phases.

Rarefaction curves (97% identity) of the bulk soil and rhizosphere samples did not reach plateau whereas rarefaction curves of cormosphere samples during two growth stages were closer to reaching a plateau indicating fungal diversity was well represented in cormosphere and would not increase on repetitive sampling (Fig 1). Comparison of rarefaction curve (97% identity) of all the samples indicated that the diversity was more in bulk soil during dormant stage as higher number of OTUs were catalogued in bulk soil in comparison to cormosphere and rhizosphere (Fig 1).This result was further complemented by the diversity indices like Chao 1 Mean and Shannon Mean (Table 2). Similar results have been reported in the study of fungal community in *Pisum sativum* wherein the diversity of fungi was more in bulk soil in comparison to rhizosphere and endorhizosphere [26]. β diversity represented by Heatmap and Jackknife tree analysis revealed a clear separation of samples according to sample type, however fungal diversity of rhizosphere and cormosphere during flowering stage were relatively similar (Figs 2 and 3) indicating niche and season specific fungi–plant associations. Similar to bacterial diversity pattern in *C*. *sativus* reported earlier [4], the fungal community is more diverse in bulk soil than the rhizosphere and least diverse in cormosphere of *C*. *sativus*.

**Table 2:** Chao1 and Shannon diversity indices of five different niches during two growth stages.

	FLOWERING STAGE			DORMANT STAGE	
Diversity indices	Bulk	Cormosphere	Rhizosphere	Bulk	Cormosphere
**Chao 1 Mean**	135.54	213.3	215.91	**220.47**	41.5
**Shannon Mean**	1.12	0.87	1.9	**2.27**	0.036

Diversity indices were maximum in bulk soil during dormant stage representing maximum fungal diversity and richness in bulk soil as compared to rhizosphere and cormosphere.

### Dynamics of fungal diversity in different niches during flowering and dormant growth stage

**Fig 4:**
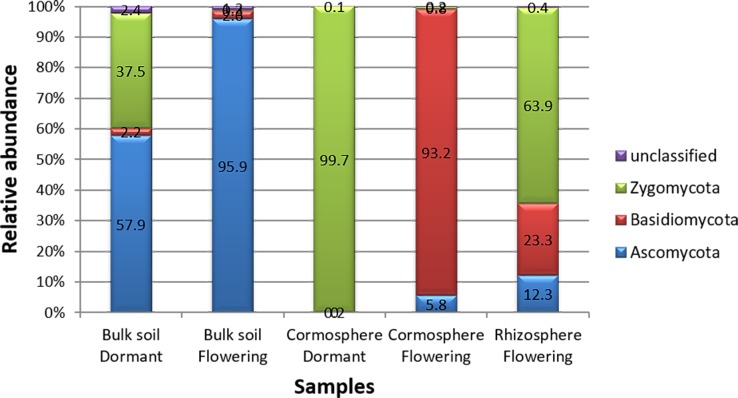
Comparison of relative abundance of fungal phyla in bulk soil, rhizosphere and cormosphere during flowering and dormant stage. The comparison indicates the dominance of *Zygomycota* in rhizosphere and cormosphere (dormant stage), *Basidiomycota* in cormosphere (Flowering stage) and *Ascomycota* in bulk soil during both stages.

Three fungal phyla namely *Ascomycota*, *Basidiomycota* and *Zygomycota* along with unclassified phylum with different richness patterns were catalogued from all the three niches during two growth stages (Fig 4). Fungal diversity associated with each niche varied significantly during the two growth stages, as is evident by the unifrac and libshuff significance value p<0.05 (S1 Table). The bulk soil was dominated by *Ascomycota* during both stages under study but, cormosphere was dominated by *Basidiomycota* in flowering stage and *Zygomycota* during dormant stage. However, rhizosphere could be studied during flowering stage only as roots are absent in the dormant stage. The rhizosphere in flowering stage was dominated by *Zygomycota*. Surprisingly, the OTU belonging to AMF could not be identified and catalogued despite analysing the sequences multiple times, although, 80% of terrestrial plants are known to have AMF associations. The reason for absence of AMF sequences in the present study is the samples analysed were soil adhering to roots (rhizosphere) and corms (cormosphere) and not root or corm tissue. Similarly, Xu and co-worker [26] has reported the fungal diversity in roots, rhizosphere and bulk soil of *Pisum sativum* by cultivation independent approaches wherein the *Glomus* a common AMF was present exclusively present in roots with very poor representation (1 OTU of *Glomus* sp) in rhizosphere samples and the bulk soil (1 OTU of *Pyrenochaeta* sp with *Glomus* sp completely absent).

As hypothesized, different fungal phyla dominated the cormosphere during two growth stages but surprisingly, the phylum that was dominant in cormosphere during one stage, was least abundant in other stage. The percentage abundance of OTU cannot be taken as real estimate of the abundance as ribosomal genes like ITS and 16S rRNA gene vary in copy number in different genera [34]. In case of 16S rRNA gene targeted metagenomic study, the data is normalized by a method that allows to improve estimation of microbial abundance and community structure by accounting for copy number variation among taxa [34]. To our knowledge such method/database have not been developed to eliminate the effect of copy number of ITS, though copy number variation in ITS has been established beyond doubt. In the present study the conclusion on the dominance/abundance are drawn if the difference is more than 25% in order to minimize the copy number effect. *Zygomycota* were abundant during dormant stage (99.7%) but almost absent (0.8%) during flowering stage whereas the abundance of *Basidiomycota* reduced from 93.2% in flowering stage to 0.05% in dormant stage (Fig 4). These results indicate the dynamic shift in fungal diversity during different growth phase in cormosphere. This shift in fungal diversity in cormosphere can be attributed to variation in the nutrients in cormosphere as well as the change in the seasonal temperature condition during two growth stages. The starch content of corm is more during dormant stage as compared to flowering stage as in flowering stage the stored resources of corm are used for flower and root development [35]. In addition, the decrease in temperature of 10–15 degree during flowering stage may be complementing reason for shift in fungal diversity.

**Fig 5:**
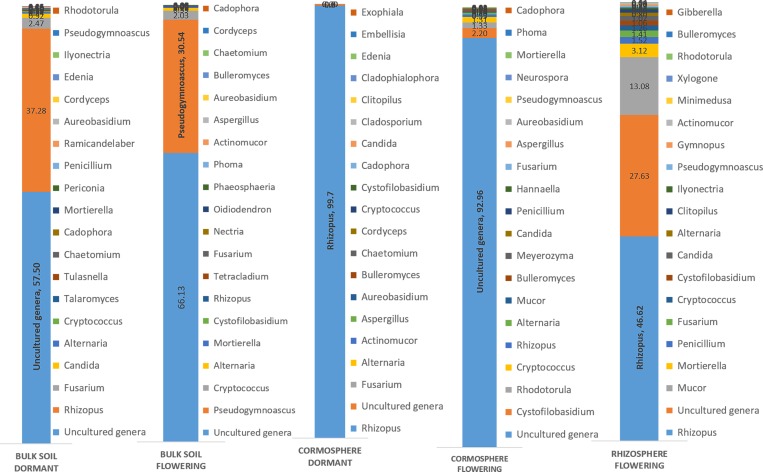
Relative abundance of fungal genera in the bulk soil, cormosphere and rhizosphere during flowering stage and bulk soil and cormosphere during dormant stage of *C*. *sativus*. During flowering stage, *Pseudogymnoascus* (30.54%) was dominant in bulk soil, *Rhizopus* (46.62%) in rhizosphere and yet–to–be–cultivated *Basidiomycota* fungi (92.6%) in cormosphere. Yet-to-be-cultivated *Ascomycota* fungi were dominant in Bulk soil = 57.50% and cormosphere = 99.7% during dormant stage.

Since there are no earlier reports available on the fungal associations of underground parts of the plants like bulb, corm, and rhizome from any plant including *C*. *sativus*, rhizosphere fungi reported in literature have been taken as a reference in the present study. Z*ygomycota* and *Basidiomycota* has been reported from the rhizosphere of Japanese barberry [21, 43] and *Pinus tabulaeformis* [44] but *Ascomycota* and *Basidiomycota* are common dominant phyla in rhizosphere [42, 44–46]. In recent years, since microbes are reported to be influencing all the biological system function from man to smallest plant, more cormosphere microbes need to unravelled for comparative analysis. However, during dormant stage, *Rhizopus* genus comprised of 99.7% of dominant *Zygomycota* phylum in cormosphere whereas yet–to–be–cultivated species of *Basidiomycota* phylum (92.6%) was dominant during flowering stage (Fig 5). *Rhizopus* has been reported from rhizospheres of red pepper [47] and have been reported to produce siderophore [48]. The production of siderophores has been implicated in the inhibition of pathogenic microbes by chelation the iron ion. This could be one of the reasons that despite being rich in starch, very few fungal types are associated with cormosphere. Dominance of yet–to–be–cultivated fungi belonging to *Basidiomycota* phylum in cormosphere during flowering stage needs to be investigated further.

**Fig 6:**
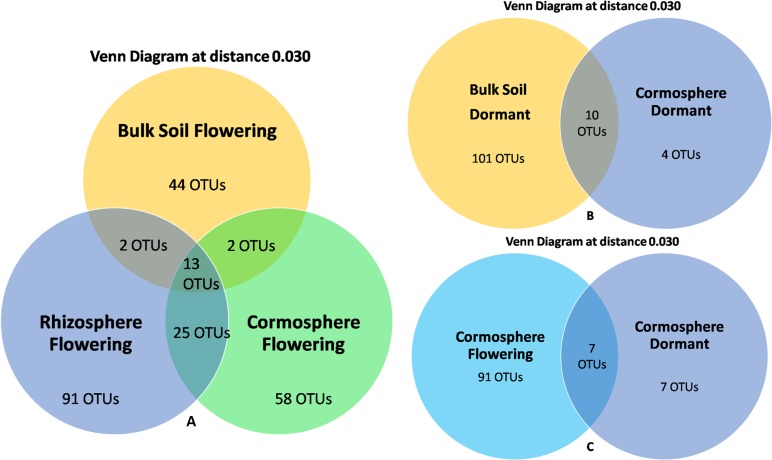
**Venn Diagrams reporting the number of OTUs shared among investigated *C*. *sativus* sample types A) bulk soil, cormosphere and rhizosphere during flowering stage. B) bulk soil and cormosphere during dormant stage C) cormosphere during flowering and dormant stage.** During flowering stage, out of total 235 OTUs, only 13 OTUs were shared by all the three niches whereas during dormant stage, only 10 OTUs were common out of 115 OTUs. Total of 105 OTUs were catalogued from cormosphere during two growth stages out of which only 7 OTUs were common.

**Fig 7:**
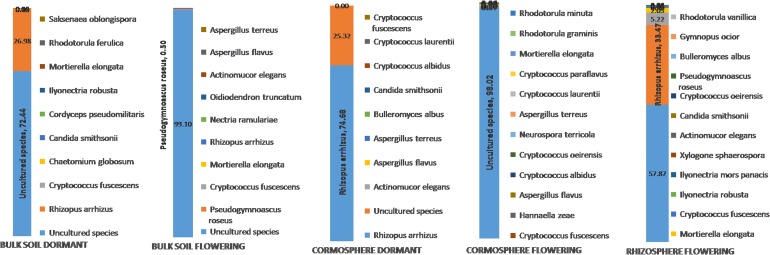
Relative abundance of fungal species in the bulk soil, cormosphere and rhizosphere during flowering stage and bulk soil and cormosphere during dormant stage of *C*. *sativus*.

A total of 105 OTUs were catalogued from cormosphere during two growth stages wherein only 7 OTUs (6.6%) were common (Fig 6C) and *Rhizopus arrhizus* was the only common species in two growth stages (Figs 3 and 7). The percentage of common fungi was less as compared to the common bacteria from cormosphere during two stages reported previously [4].

The fungal diversity of *C*. *sativus* rhizosphere could not be compared during two growth stages due to the absence of roots in dormant stage. In flowering stage, the fungal diversity of rhizosphere was dominated by *Zygomycota* (63.9%) followed by *Basidiomycota* (22.3%) and *Ascomycota* (12.3%) (Fig 4). Contrary to present study, rhizosphere fungal community is reported to be usually dominated by saprotrophytic fungi of phyla *Ascomycota* and *Basidiomycota* [30, 42, 44–46]. *Zygomycota* have also been reported from rhizosphere e.g. Japanese barberry, *Pisum sativum*, maize, red pepper and tomatoes [21, 26, 30, 43, 47, 48] though in low abundance. *Zygomycota* phylum were dominant in flowering rhizosphere and dormant cormosphere (Fig 4), but in cormosphere the phyla is represented only by *Rhizopus* (99.7%) whereas in flowering rhizosphere though *Rhizopus* (46.62%) was the dominant genus but other genera *Mucor* (13.08%) and *Moritiella* (3.12%) also contributed to the community (Fig 5, S2 and S3 Tables).

Bulk soil, though dominated by *Ascomycota* in both stages, varied in its abundance (95.9% during flowering and 57.9% during dormant stage). During dormant stage *Zygomycota* (37.5%) was also abundant in bulk soil which otherwise was rare during flowering stage (0.3%) (Fig 4). The shift in representation of *Zygomycota* from flowering stage to dormant stage is similar to cormosphere where in *Zygomycota* is the second dominant phyla in dormant stage and rare during flowering stage. This variation in bulk soil fungal community during two growth stages was also represented by different clades of bulk soil in Jackknife dendrogram (Fig 2). The abundance of *Basidiomycota* did not change much during the two stages i.e. 3.3% and 3.6% (Fig 4). Abundance of *Ascomycota* in bulk soil is also reported in *Pisum sativum* and forest bulk soils [26, 42]. During flowering stage, the abundance of fungal genera *Pseudogymnoascus* and *Pseudogymnoascus roseus* species belonging *Ascomycota* phylum was observed whereas during dormant stage, the *Ascomycota* phyum, being dominant, could not be classified up to genus or species level due to absence of reference sequences in databases (Figs 5 and 7). *Pseudogymnoascus* abundant in the bulk soil in present study has been also reported from grass rhizosphere [49].

*Fusarium oxysporum* is reported to be pathogenic to *C*. *sativus* causing corm rot that leads to great loses in its produce. The incidence of corm rot in *C*. *sativus* grown in Kashmir is about 70–80% [50] but no parallel study on microbial diversity of diseased saffron corm using cultivation independent approach has been done so far. *Fusarium* was present in all the soil type and, since the OUT abundance is being compared within same genera associated with different belowground parts, therefore the copy number of ITS here would be more or less similar (Bulk soil flowering = 0.05%, Bulk soil flowering = 1.4%, Cormosphere flowering = 0.06%, Bulk soil dormant = 2.47% and Cormosphere dormant = 0.05%). Surprisingly, the abundance of *Fusarium* was low in cormosphere and did not show much variation in abundance during dormant and flowering stage (S2 and S3 Tables). The samples were collected from the a field in Wuyan, Kashmir, J&K where the incidence of corm rot was comparatively low which could be the reason for overall low abundance of *Fusarium* genera. However during flowering it was concentrated in roots and in dormant stage it was concentrated in soil and not the corm. The OTUs belonging to *Fusarium* could not be classified upto species level due to lack of the sequence homology with the reference sequences available in the various databases (Fig 5, S2 and S3 Tables) therefore the distinction between pathogenic and non–pathogenic *Fusarium* could not be drawn. Though corm has been cited as the site of infection for fungal penetration and in in-vitro experiments pathogenic *Fusarium oxysporum* has been shown to infect the corm both in plate and pot assays [50] however in the present study, the low abundance of *Fusarium* sp on corm in comparison to root is interesting to study further.

**Fig 8:**
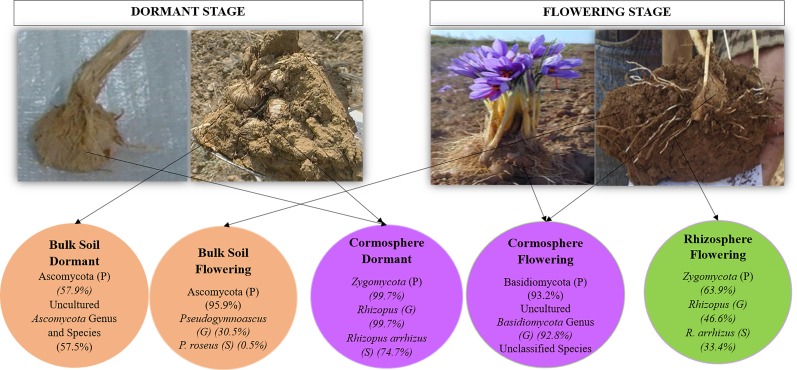
Dominance pattern of fungal community in each of niche during two growth stages. During flowering stage, dominance of *Rhizopus arrhizus (Zygomycota* phylum) in rhizosphere, *Pseudogymnoascus roseus (Ascomycota* phylum) in bulk soil was observed whereas in the cormosphere, the sequences belonging to dominant *Basidiomycota* phylum could not be classified upto genera or species level. During dormant stage, *Rhizopus arrhizus (Zygomycota* phylum) was dominant in cormosphere whereas in the bulk soil the sequences belonging to dominant *Ascomycota* phylum could not be classified upto genera or species level. In the figure, P represents phylum, G represents genus and S represents species of fungi.

In addition to seasonal (temporal) variations, niche specific (spatial) variations were also observed in a particular growth stage of *C*. *sativus* as indicated by dominance of different fungi in different niches during flowering stage and similar pattern was observed in dormant stage (Figs 5 and 8). A total of 90 fungal genera were catalogued from all the niches during two stages but only 45 genera could be identified up to species level due to lack of the sequence homology with the reference sequences available in the various databases, (S2 and S3 Tables). During flowering stage, *Rhizopus arrhizus (Zygomycota* phylum) was dominant fungal species in the rhizosphere whereas *Pseudogymnoascus roseus (Ascomycota* phylum) was dominant in bulk soil thereby indicating niche specific variations in a particular growth stage. However, in the cormosphere, the sequences could not be classified upto genera or species level. Although dominance of different fungal species were observed across different niches, there were few species which were common in all the three niches. Out of total 235 OTUs, only 13 OTUs (5.53%) were shared by all the three niches (Fig 6A). At species level, only 3 species namely *Cryptococcus fuscescens*, *Mortierella elongata* and *Rhizopus arrhizus* were common in the three niches (Fig 7). Similar variations were observed during dormant stage also wherein out of total 115 OTUs, only 10 OTUs (8.69%) were common (Fig 6B) and only a single species, *Rhizopus arrhizus (Zygomycota phylum)* was common between bulk soil and cormosphere during dormant stage (Fig 7). These finding were correlating to previous study of niche specific bacterial associations [4].

The fungal associations to the various belowground parts of *C*. *sativus* are not only organ specific but are growth stage specific. These finding are in agreement to our previous study where we have reported similar organ specific association pattern for bacteria [4]. We propose that fungal community associated with *C*. *sativus* is also dynamic and are constantly changing in response to the availability of nutrients and development / growth stage. Since this is the first report of its kind and no reference is available from J&K India or anywhere in the world to draw conclusion. Further investigation of the mycobiome of belowground parts of *C*. *sativus* from various site from all over the world need to be studied in future.

As per their pattern of dominance present study suggests *Rhizopus* in the rhizosphere can be promising candidates to be cultivated and characterized for plant growth promotion activities and future use as PGPF (Plant Growth Promoting Fungi).

## Conclusion

Rhizosphere is the most studied microbial niche in the plants but restricting the investigation to only roots will expose the tip of the iceberg, hence the microbial association with all the parts of plant need to be studied to have a comprehensive picture. Environmental genomics has proved beyond doubt that microbes are associated with all the living being and play important role for their survival and well being. This spatial and temporal dynamic of the plant–microbe interaction needs to be studied extensively in various plants covering individual organ of each plant. This study, on the microbial association with *C*. *sativus* rhizo- and cormosphere, in agreement with previous studies that have suggested that not only are these association organ specific but they vary during the various growth stages. However the effect of seasonal variations on fungal community dynamics due to change in weather cannot be ignored. Knowledge of such interactions in the rhizosphere and cormosphere can help in relating microbial community changes to environmental variations over time and its effect on plant health.

## Supporting Information

S1 Table Significance values based on libshuff and Unifrac weighted analysis. (XLSX)

S2 Table Abundance of fungal (phylum, genus and species) diversity in Bulk soil, cormosphere and rhizosphere of C.sativus during flowering stage. In the table, P represents phylum, g represents genus and s represents species of fungi
(XLSX)

S3 Table Abundance of fungal (phylum, genus and species) diversity in Bulk soil and cormosphere of C.sativus during dormant stage. In the table, P represents phylum, g represents genus and s represents species of fungi
(XLSX)
